# Removal of Ovarian Cyst, Hematoma and Fibroid

**Published:** 1902-05

**Authors:** Francis W. Barton

**Affiliations:** Interne


### CLINICAL REPORTS.

### Removal of Ovarian Cyst, Hematoma and Fibroid.

By L. G. HANLEY, M. D.,
Gynecologist Buffalo Hospital of the Sisters of Charity.
Reported by FRANCIS W. BARTON, M. D., Interne.

MISS D., age 36; has been treated for stranguary during the past year. Was obliged to urinate every three or four hours. The annoyance caused by irritation from bladder was of such severity, that she became at times hysterical, irritable and somewhat unbalanced mentally. Patient has suffered for two years with severe dysmenorrhea. Examination revealed a complete retroversion of the uterus, with a mass on either side showing evidence of some inflammatory action.

Patient was prepared for celiotomy. On opening the abdomen the following conditions were found, viz.: cyst of the right ovary the size of a small orange, hematoma of the left ovary two inches in diameter, and a subperitoneal fibroid, lying one-half inch below and posterior to the fundus of the uterus. Adhesions were broken up and specimens removed, the uterus anchored to the abdominal wall according to the method described by Dr. Hanley in the February number of the Buffalo Medical Journal. Stitches were removed on the tenth day. Patient sat up on the seventeenth day. Has complained of no bladder symptoms since operation, and feels perfectly well.

This case was unique, owing to the presence of an ovarian cyst, hematoma and a fibroid, in the same patient, and in so far as the mental disturbance was relieved by the operation.

